# Semantic descriptor ranking: a quantitative method for evaluating qualitative verbal reports of visual cognition in the laboratory or the clinic

**DOI:** 10.3389/fpsyg.2014.00160

**Published:** 2014-03-04

**Authors:** Matthew Maestri, Jeffrey Odel, Jay Hegdé

**Affiliations:** ^1^James and Jean Culver Vision Discovery Institute, Georgia Regents UniversityAugusta, GA, USA; ^2^Brain and Behavior Discovery Institute, Georgia Regents UniversityAugusta, GA, USA; ^3^Department of Ophthalmology, Columbia University College of Physicians and SurgeonsNew York, NY, USA; ^4^Department of Ophthalmology, Medical College of Georgia, Georgia Regents UniversityAugusta, GA, USA

**Keywords:** qualitative research, natural language processing, semantic processing, visual cognition, neuropsychological tests

## Abstract

For scientific, clinical, and machine learning purposes alike, it is desirable to quantify the verbal reports of high-level visual percepts. Methods to do this simply do not exist at present. Here we propose a novel methodological principle to help fill this gap, and provide empirical evidence designed to serve as the initial “proof” of this principle. In the proposed method, subjects view images of real-world scenes and describe, in their own words, what they saw. The verbal description is independently evaluated by several evaluators. Each evaluator assigns a rank score to the subject’s description of each visual object in each image using a novel ranking principle, which takes advantage of the well-known fact that semantic descriptions of real life objects and scenes can usually be rank-ordered. Thus, for instance, “animal,” “dog,” and “retriever” can be regarded as increasingly finer-level, and therefore higher ranking, descriptions of a given object. These numeric scores can preserve the richness of the original verbal description, and can be subsequently evaluated using conventional statistical procedures. We describe an exemplar implementation of this method and empirical data that show its feasibility. With appropriate future standardization and validation, this novel method can serve as an important tool to help quantify the subjective experience of the visual world. In addition to being a novel, potentially powerful testing tool, our method also represents, to our knowledge, the only available method for numerically representing verbal accounts of real-world experience. Given that its minimal requirements, i.e., a verbal description and the ground truth that elicited the description, our method has a wide variety of potential real-world applications.

## INTRODUCTION

In real-world situations, our perception of visual scenes tends to be complex and nuanced, with rich semantic content. Capturing this complexity is critical not only for the study and treatment of visual dysfunction, but also for the study of normal visual function. For practical reasons, the available quantitative tests of visual perception tend to use relatively simple visual stimuli and tasks that constrain the responses of the test subject (e.g., contrast sensitivity test, line bisection test, star cancellation test), so that the responses can be precisely measured and quantitatively analyzed (Green and Swets, 1974; Gescheider, 1997; Lezak, 2012).

The importance and usefulness of traditional quantitative tests in research and clinical settings is indisputable. But it is also clear that quantitative tests of visual perception have a major drawback, in that they fail to capture the complexity of visual function and dysfunction in real life. That is, the complex, qualitative nature of normal high-level visual perception under real life conditions is all but impossible to measure using the available quantitative tests. Impairments of high-level visual perception are similarly, hard to measure.

At the other end of visual testing spectrum, qualitative tests of visual function have a roughly complementary set of strengths and weaknesses, in that while they are much better at capturing the nuances of high-level vision under real-world conditions, the outcomes of these tests are hard to quantify (Miles and Huberman, 1994; Poreh, 2000; Ogden-Epker and Cullum, 2001). Imagine, for instance, a clinical provider trying to quantify the visual deficit in a patient with agnosia, or inability to recognize objects. A typical test is to show the patients drawings of everyday objects, such as a pen, mug etc., and ask them to redraw and name it. Patients with a clear-cut apperceptive agnosia fail both to draw and name the object, whereas patients with clear-cut associative agnosia generally are able to draw the object, but not to name it. Even when the outcome of the test is clear-cut as this, it is hard to measure the quality and the completeness of the drawings and naming. Moreover, the actual clinical outcomes are rarely as clear-cut, with most patients showing symptoms that cannot be neatly pigeonholed into either of the above two extremes (Atkinson and Adolphs, 2011; Barton, 2011; Mitchell, 2011). Furthermore, the outcomes of this test are affected by an array of complexities of agnosia. Thus, while the test outcomes are rich in qualitative information, it is hard to measure this information. This is a well-documented shortcoming of qualitative tests in general (Gainotti et al., 1985, 1989; Milberg et al., 1996; Glozman, 1999; Ogden-Epker and Cullum, 2001; Pachalska et al., 2008).

Quantifying qualitative reports would effectively meld the best of both worlds, by combining the ability of the qualitative methods to capture the richness of the visual experience in the real-world with the scientific rigor of the quantitative methods. A large number of such methods have been developed, with applications in clinical care, educational testing, machine learning and scientific research (for reviews, see Udupa, 1999; Auerbach and Silverstein, 2003; Gustafson and McCandless, 2010; Sauro and Lewis, 2012; Bazeley, 2013). While a review of this large and diverse literature is beyond the purview of the present report, two aspects of the quantification process are particularly worth noting. First, the existing methods generally require that the qualitative report be formatted or structured (e.g., questionnaires), so as to streamline the quantification process. That is, the underlying qualitative reports are generally not open-ended. Second, to our knowledge, no methods exist in clinical, psychophysical or machine learning literature for creating a numeric representation of verbal reports. This latter issue is particularly relevant when dealing with real-world visual percepts, which have a rich semantic content (Miles and Huberman, 1994; Glozman, 1999; Poreh, 2000; Zipf-Williams et al., 2000; Joy et al., 2001; Ogden-Epker and Cullum, 2001).

In this report, we propose a novel methodological principle that will help address both of the aforementioned shortcomings of the currently available approaches, and is well suited to complement (albeit not replace) the rich array of available methods. Our method, which we will refer to as semantic descriptor ranking (SDR), allows quantification of open-ended, verbal reports of visual scenes. We illustrate its implementation using perceptual reports of complex real-world scenes by healthy subjects. As noted above, the present report only aims to provide a proof of concept of the proposed method, i.e., that the proposed method is feasible. Our implementation will also help highlight issues involved in the future development and refinement of the proposed method, including its standardization and validation (Glozman, 1999; Ogden-Epker and Cullum, 2001; Chiappelli, 2008; Salkind, 2010).

## MATERIALS AND METHODS

### PARTICIPANTS

Fourteen different volunteer adults (6 females) participated in one or both of the two experiments that constituted this study. Subjects were 19 to 31 years of age (median age, 24 years). In either experiment, some participants participated as subjects who viewed the stimuli and reported their percepts, and others participated as evaluators, who scored the subjects’ reported percepts. No one participated both as a subject and as an evaluator. That is, no one who participated in either experiment as a subject also participated in either experiment as an evaluator, or *vice versa*. All subjects had normal or corrected-to-normal vision, with no known neurological or psychiatric disorders.

Experiment 1 consisted of six subjects and two evaluators, and Experiment 2 consisted of eight subjects and two evaluators. All participants gave informed consent prior to participating in the study. All protocols used in the experiment were approved in advance by the Human Assurance Committee at the Georgia Regents University, where this study was carried out.

### VISUAL STIMULATION

**FIGURE 1:**
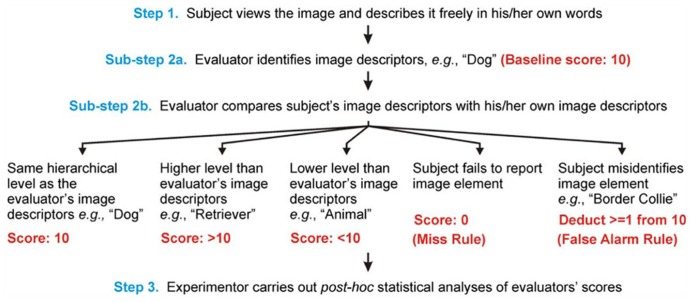
**Workflow of SDR.** The three main steps of SDR, which involve obtaining, scoring and analyzing the subject’s reports respectively, are shown. Note that the subject, the evaluator, and the experimenter plays the most prominent role in Steps 1, 2, and 3, respectively. The two sub-steps of Step 2 in which the evaluator scores the subject’s reported percept are illustrated here using a hypothetical exemplar image (not shown) in which one of the image elements is a dog. Sub-steps 2a and 2b are repeated for each image element in each image (not shown). See text for details.

In Experiment 1, 50 different real-world photographs from the Corel Stock Photo Library (Corel Corporation, Ottawa, ON, Canada) were used as visual stimuli in this study (see, e.g., **Figures 1** and **3**). Subjects sat comfortably approximately 30 cm in front of a computer monitor in a normally lit room (ambient luminance, 14.6 cd/m^2^). Each trial started when the subject indicated readiness by pressing a button. The visual stimulus was presented for 50 ms or 17 ms, depending on the condition, followed by a random dot mask (**Figure 1**). These two stimulus durations correspond to 1 or 3 frame durations of the computer monitor at a screen refresh rate of 60 Hz.

Trials were presented in a pseudo-random order. To minimize the contribution of stimulus repetition on the subject’s reports (Ochsner et al., 1994; Maxfield, 1997; Gauthier, 2000; Henson, 2003; Holcomb and Grainger, 2007; Kristjansson and Campana, 2010), we ensured that the 50 ms viewing of a given stimulus preceded its 17 ms viewing within the pseudo-random sequence of trials.

The stimulus subtended 9° × 6° (for landscape format pictures; the reverse for portrait format pictures), and had an average luminance of 30.2 cd/m^2^, and was presented against a uniform gray screen of the same mean luminance. The mask had the same average luminance and subtended 9° × 9°. Following the mask, the subject had unlimited time to orally describe, in his/her own words and with no prompting or feedback, what he/she saw in the visual stimulus. The description was audio-recorded.

Experiment 2 was identical to Experiment 1 except that it used a different, non-overlapping set of 50 images, and a different, but partially overlapping set of subjects and evaluators.

### RATIONALE BEHIND SDR

Semantic descriptor ranking takes advantage of the fact that our semantic understanding, and therefore the reported percept, of visual objects tends to have a naturally hierarchical structure: A large number of previous studies have shown that our understanding of real-world objects generally (although not always, see Discussion) follows a hierarchical pattern of categories (Rosch, 1973; Hanson and Hanson, 2005; Hegdé, 2008). For instance, a particular pet dog named “Spike” can be thought of, in an order of increasingly finer categorization, as an object, an animal, a mammal, a dog, a retriever, a Golden retriever, and finally as a particular dog named Spike. This hierarchical organization lends itself to ranking, so that the above descriptors can be rank-ordered, in increasing order of specificity, as object < animal < mammal < dog < retriever < Golden retriever < Spike. Similarly, “brown dog” can be reasonably considered a more specific, and therefore higher ranking, description than “dog”. These ranks can be analyzed using the established rank-based statistical methods.

Given that ranking semantic “tags,” or descriptors, is central to our method, we refer to it as SDR. We use the term “semantic descriptor” to mean a word or phrase (i.e., a verbal “tag”) that describes a given object, to distinguish it from the term “[image] descriptor” commonly used in machine vision, which generally refers to various lower-level properties of the image, such as color, texture, or local shape (Lowe, 1999; Mikolajczyk and Schmid, 2001; Belongie et al., 2002; Manjunath et al., 2002; Pollefeys and Gool, 2002; Lazebnik et al., 2005; Snavely et al., 2006).

### IMPLEMENTATIONS OF SDR: VARIATIONS OF A THEME

A typical implementation of SDR would consist of the following three steps, in order (see **Figure 1**; also see below): (1) Subjects freely view pictures of real-world scenes and describe in their own words what they see. (2) A set of independent evaluators examine each subject’s reports and rank the descriptors according to how specific the descriptors are. Since each descriptor will be assigned a rank score, the report as a whole will typically consist of multiple rank scores. Collectively, these rank scores are a numeric representation of the verbal report. (3) The experimenters analyze the numeric representations using conventional statistical methods.

Note that a large number of variations of the above theme are possible; one can customize SDR for a given purpose by appropriately varying one or more of the above three steps. Indeed, the only two crucial requirements of SDR are that (a) the reports be verbal (i.e., spoken or written), (b) the image or scene underlying the report be available for independent evaluation (i.e., the evaluator be able to see what the subject is seeing).

With these minimum requirements met, one can create a numeric representation of a given perceptual report of interest (“query representation”) and appropriately compare it to a reference of some sort. Note that this reference can be arrived at by any of a large number of possible principled methods. For instance, the reference representation can be obtained using the same subject viewing the same image under a different viewing condition (e.g., different stimulus duration, see below). For a hemineglect patient, for instance, the query and reference representations can be obtained using stimulus presentations in the affected and spared hemisphere, respectively. Each of these instances makes for a two-sample, within-subject paired design, where the query- vs. reference representations constitute the two samples. Alternatively, one can use a one-sample design, where the query representation from one subject can be compared against an existing reference sample obtained from, say, a large number of other subjects. Note that the query and/or reference representations can, in principle, be obtained using machine vision algorithms, rather than human subjects (see Discussion).

## RESULTS

### AN ILLUSTRATIVE IMPLEMENTATION AND PROOF OF PRINCIPLE OF SDR

We will illustrate the use of SDR using a two-sample, within-subject paired design that compared the verbal reports of each given subject on the same set of images using two different stimulus durations. This design exploits the previously known fact that, in general, longer viewing of visual stimuli elicits finer-grained perception than briefer viewing (Sugase et al., 1999; Liu et al., 2002; Grill-Spector and Kanwisher, 2005; Hegdé, 2008; also see Discussion).

We carried out two experiments. Experiment 1 compared the reports elicited by the viewing of the same set of real-world scenes for long vs. brief durations (50 ms vs. 17 ms, respectively; see Materials and Methods for details). It tested the hypothesis, using SDR, that the responses elicited by the 50 ms viewing collectively will have higher rankings than the responses elicited by the 17 ms viewing.

### STEP 1: OBTAINING QUALITATIVE REPORTS FROM THE SUBJECTS

**FIGURE 2:**
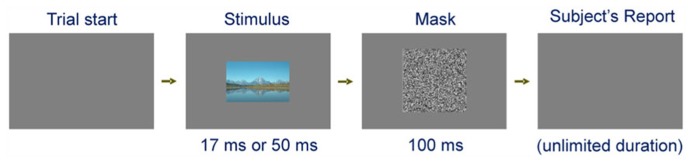
**Trial paradigm used in our implementation of Step 1.** Each trial started when the subject indicated readiness. The visual stimulus (a real-world image) was presented for 17 ms or 50 ms, depending on the trial. To minimize the contribution of stimulus repetition on the subject’s reports, each given image was presented for the longer stimulus first, as described in Materials and Methods. After the 100 ms mask, the subject was allowed unlimited time to describe, in his/her own words, what he/she perceived in the stimulus. The figure is not drawn to scale.

Subjects viewed natural images one per trial, presented for either 50 ms or 17 ms, depending on the trial (**Figure 2**; see Methods for details). After a brief mask, the subjects were allowed unlimited time to describe, in their own words and *ad libitum*, what they saw in the stimulus. The subjects’ reports were audio-recorded.

Each subject viewed each image twice, first for the longer stimulus duration and then for the shorter duration, in blocks of randomly interleaved trials (see Methods). The rationale for always first viewing the image for the longer duration was to minimize the contributions of priming/exposure effects, where a previously viewed stimulus tends to elicit better recognition during subsequent viewing (Ochsner et al., 1994; Maxfield, 1997; Gauthier, 2000; Henson, 2003; Holcomb and Grainger, 2007; Kristjansson and Campana, 2010). Note that this meant that the priming/exposure effects would actually tend to counteract, i.e., reduce, the expected increase in rankings upon longer stimulus viewing. Thus, our method would have to find duration-dependent effects, if any, over and above the counteracting effects of priming.

### STEP 2. INDEPENDENT RANKING OF THE SUBJECT’S REPORTS BY EVALUATORS

This step essentially consisted of ranking, separately by each of the evaluators, of the descriptors used by subjects in their oral report. This is the crucial step in SDR, in which qualitative reports of the subjects are converted into quantitative measures.

**FIGURE 3:**
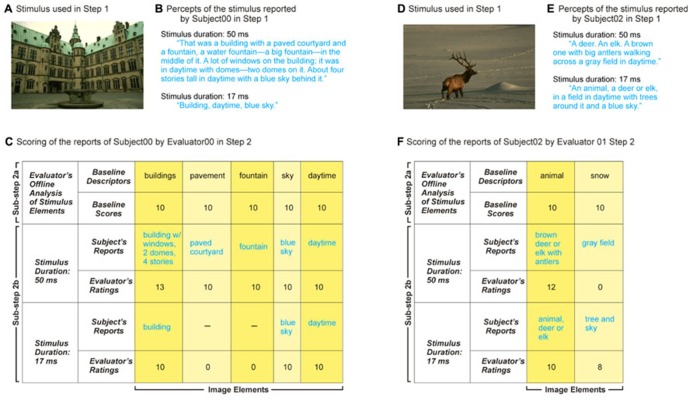
**Two instances of implementation of SDR steps 1 and 2.** Panels **A** through **C** show the scoring of one set of verbal reports, and panels **D** through **F** show the scoring of a second, independent set. **(A)** Stimulus. Subject viewed the stimulus for 50 ms and 17 ms respectively in randomly interleaved trials, so that subject reports were paired across the experiment. Note even though each subject viewed the same stimulus twice, the longer viewing duration always preceded the shorter viewing duration, so as to counteract priming effects, if any (see text for details). **(B)** Percepts of the stimulus in panel **A** as reported by a subject for each of the two stimulus durations. **(C)** Scoring of the subject’s reports in panel **B** by the evaluator. Note that, although the subject’s descriptions of the building were spread over multiple sentences, the evaluator grouped them together into a single descriptor, in accordance with the scoring rules. Columns corresponding to various image elements are highlighted in different colors solely to enhance visibility. **(D–F)** Scoring of a different pair of reports.

Before the evaluations began, the evaluators received extensive training in the relevant procedures. In addition to the routine scoring procedures (outlined in **Figures 1** and **3**), we devised a set of somewhat arbitrary, but principled, evaluation rules for handling special cases (some of which are shown in **Table 1**) in order to help ensure that these cases were handled as consistently as possible. Note that the evaluation rules can be customized for each given application of SDR. Note also that it is possible, in principle, to write computer programs to automate the evaluation process.

Each subject’s reports were scored by multiple evaluators independently of each other and of the subject. The scoring process consisted of two sub-steps (**Figures 1** and **3C,F**): Sub-step 2a consisted of the evaluator’s own offline analysis of each image prior to evaluating the subject’s reports, in which each evaluator viewed each given image *ad libitum*, and wrote down any number of semantic descriptors as he/she thought were needed to capture what was in the image. Each descriptor was assigned an arbitrary baseline value of 10. It is important to emphasize that the absolute value of the baseline score (or of other scores for that matter, see below) is unimportant; any value that allows sufficient room for deductions and bonuses (i.e., sufficient spread from the baseline) will suffice. That is, what matters in our particular implementation of SDR are the *relative* scores, rather than the *absolute* scores, since our implementation ultimately uses rank statistics (see Discussion for other implementation options). For the same reason, the absolute hierarchical level of the descriptor (“Dog” vs. “Golden retriever”) that a given evaluator comes up with [which, among other things, depends on the expertise of the evaluator (Rosch, 1973; Palmeri and Gauthier, 2004); also see Discussion] does not matter in the present context either.

In Sub-step 2b, the evaluators listened to the audio recording of the subject’s perceptual report of the same stimulus, and scored the subject’s descriptions of the image *relative to the evaluator’s image descriptors from Sub-step 2a* according to a set of pre-specified rules (see **Table 1**). If the subject’s descriptor was deemed to be essentially the same as the corresponding descriptor of the evaluator (e.g., “dog”), the subject’s report for the given image descriptor was also assigned the baseline value.

**Table 1:** Selected special case rules.

Rules
1. Objects (i.e., nouns, such as “dog”) are primary descriptors, while adjectives/modifiers such as colors (e.g., “black”) are secondary descriptors. Descriptions with correct primary and secondary descriptors should receive higher ranking than descriptions with a correct primary descriptor but without a secondary descriptor.
2. If the primary descriptor is correct, but the secondary descriptor is wrong, award the appropriate points for the correct primary descriptor, and simply ignore the incorrect secondary descriptor, but do not deduct points for it.
For example, if the stimulus contains a red car, and the subject’s report describes a red car, then award plus a bonus point for the correct secondary identifier. But if the subject reports a blue car, simply take the bonus points away, but do not deduct from the point you were going to award for the correct primary descriptor. The reason for this rule is to ensure that, in the above case for instance, “blue car” does not receive fewer points than simply “car.”
3. Miss Rule. If an object is present in the image, but it is not reported, then award a score of 0 for that descriptor.
4. False Alarm Rule. If an object that is not present in the image is reported, then assess a penalty of -1. For example, if the subject reports a car when, in fact, there is no car in the picture, then the score should be reduced by 1. Also assess a penalty if an object is reported as something else entirely. For example, the image contains a tree and the subject reports a building instead of a tree then a penalty of -1 should be assessed.
5. If there is more than one object of the same kind (e.g., more than one person) award a bonus of +1 for each additional person recognized. However, there is no penalty if the subject does not report all the persons in the image. The following are just two examples and could apply for any type of objects.
Example 1: An image has three dogs. The subject reports three dogs. The score should be 10 + 1 + 1 = 12. Default score of 10 for one recognized and 1 point added per dog.
Example 2: An image has three dogs. The subject reports one dog. Then it is still rewarded the standard 10 for recognition of a dog, and no penalty for not identifying the rest.
6. In those cases where the secondary descriptor is redundant with the primary descriptor (e.g., “blue sky,” “green grass”) do not award extra points for the secondary descriptor. When the secondary descriptor is not redundant (e.g., the stimulus contains brown grass), award bonus points for correct secondary descriptor (in this case, “brown”).

If the subject’s description was more specific (“Golden retriever”) than that of the evaluator, the subject’s description was assigned a correspondingly higher score. The exact decrement or increment of the score was up to the evaluator, but he/she was required to be consistent about it across subjects. For instance, “Golden retriever” can be reasonably considered one, or two, ranks higher in terms of the level of categorization than “Dog”, depending on whether the evaluator recognizes an intermediate category of “Retriever.” Similarly, if the subject’s descriptor was less specific (“animal”) than the evaluator’s corresponding descriptor (“dog”), the subject’s report was given a correspondingly lower score.

If the subject failed to report a given object altogether, the given image descriptor was assigned a value of 0 (“Miss Rule” in **Figure 1**; also see **Table 1**). If the subject misidentified an image element (e.g., when a Golden retriever was identified as “Border Collie”), the subject was penalized one or more points according to the hierarchical level of the reported identifier (“False Alarm Rule”). That is, the subject was awarded the appropriate score for having recognized that it was a dog, and was then penalized 1 point for misidentifying the breed. Note that while this is a somewhat arbitrary rule, it is also principled, and has considerable precedence (Green and Swets, 1974; Geissler et al., 1992). Note, in any event, that the drawbacks of our implementation, such as they may be, are not the drawbacks of SDR *per se*. Investigators can take advantage of the basic SDR principle but nonetheless devise their own set of implementation rules.

An actual image used in Experiment 1 is shown in **Figure 3A**. The reports of one subject after viewing it for 50 ms and 17 ms are shown in 3B. The corresponding scoring of a typical evaluator scored the two reports using our scoring method is shown in **Figure 3C**. Note that, as expected, the report for longer duration elicited ratings equal to or better than, the baseline scores for all image identifiers, whereas with the shorter duration, the subject missed a few image identifiers. Thus, the scoring method did reveal that longer viewing also produced a finer-grained percept of the image. **Figures 3D–F** illustrate the reports of another subject of a different image and the corresponding scores assigned by a different evaluator. The scores were lower for the shorter image duration, because the subject misidentified the snowy background in this case.

**FIGURE 4:**
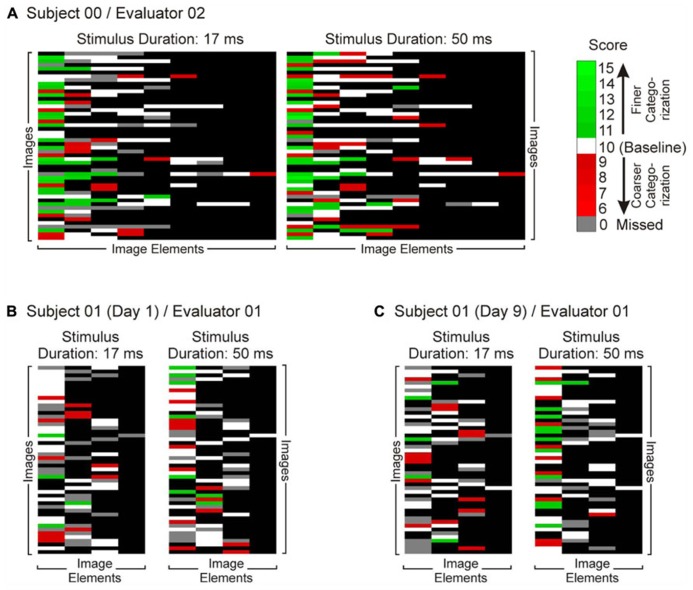
**Comparison of subjects’ reports for the short (17 ms) and long (50 ms) stimulus durations.** Subjects viewed images for either stimulus duration, and reported their percepts using the paradigm illustrated in **Figure 2**. Subjects’ reports were scored by an evaluator as illustrated in **Figures 1** and **3**, and the resulting scores are shown in this figure in color-coded fashion. Panels **A** though **C** show data from three different, representative data sets, each obtained using the same set of images. Each row in each panel shows the numeric representation of a single verbal report. Rows are matched across panels **A–C**, so that, for instance, row 7 in each panel denotes reports of the same image by different subjects and/or during different sessions. Each column denotes a different descriptor. Note that the columns are not necessarily matched across panels **A–C** (although they’re exactly matched within each panel), because the subjects did not necessarily describe the same set of image elements even though the underlying images were the same. The order of rows or columns has no particular meaning, so that the only meaningful comparison is between paired cells within each data set. All data are rendered on a black background according to the color scale at top right. White cells denote image elements for which the subject used the same descriptor as the baseline descriptor set by the given evaluator. Green and red hues denote image descriptors that were, respectively, more specific or less specific than the evaluator’s baseline descriptors. Gray cells denote the descriptors the subject used during one viewing, but omitted during the other. **(A)** Reports of Subject 00 as scored by Evaluator 02. Since this particular subject reported í9 descriptors for any given image, there are nine columns in this data set. **(B)** and **(C)** denote the reports of Subject 01 in two successive, duplicate sessions 9 days apart, as scored by same evaluator (Evaluator 01). See text for details.

Finally, to help account for individual differences across subjects and evaluators, we repeated the first three steps independently across multiple subjects and evaluators (Reynolds and Willson, 1985; Miles and Huberman, 1994; Milberg et al., 1996; Poreh, 2000; Ogden-Epker and Cullum, 2001). Some of the representative results are shown in **Figure 4A** in a color-coded format. In general, subjects’ reports for the longer stimulus duration elicited larger scores than their reports of the same image for the shorter viewing duration, as denoted by the fact that there were a greater number of descriptors and more of the descriptors had higher-than-baseline values (i.e., greener cells) in **Figure 4A**.

### STEP 3: *Post hoc* STATISTICAL ANALYSES OF THE EVALUATORS’ SCORES

We tested each numerical score produced by the evaluators using the conventional paired two-sample Mann–Whitney test. As noted above, based on previous studies, we expect *a* *priori* that longer stimulus durations produce finer-grained percepts (Liu et al., 2002; Grill-Spector and Kanwisher, 2005; Xu et al., 2005; but see Mack et al., 2008, 2009). For each of the three subjects and either evaluator, 50 ms viewing of the images did elicit significantly finer-level categorization (one-tailed paired Mann–Whitney, *p* < 0.05 in all cases).

To test the reproducibility of the results, we re-tested one subject after a 9-day delay, so as to minimize priming or other memory-related effects from the first session. The scores of the two sessions are shown in **Figures 4B,C**. The scores were statistically indistinguishable between the two sessions (2-way ANOVA, session × stimulus duration; *p* < 0.05 for stimulus duration factor and *p* > 0.05 for session and interaction factors).

The scores were also consistent between the two evaluators across all datasets (Cronbach’s alpha test, α = 0.87; data not shown). Thus, the scores did not significantly depend on the particular evaluator used.

A principled validation method for the scoring algorithm is to test whether the scores can predict the corresponding stimulus condition. The underlying rationale is that if the numerical scores of the evaluators reliably reflect the reports, and the reports in turn are a reliable reflection of the stimulus duration, then it should be possible to predict the stimulus duration based on the corresponding scores. We found this to be true for all six data sets (Spearman rank correlation, *r* ≥ 0.67, *df* = 49 for all six data sets; data not shown), indicating that the scores reliably reflect the underlying stimulus conditions.

We obtained qualitatively similar results in Experiment 2, which used a different set of images, subjects and evaluators, compared to those used in Experiment 1 (data not shown). Together, the results of the two experiments indicate that our above results are not idiosyncratic to the stimuli, subject and evaluators used. Our results also indicate that SDR is a sensitive technique that can detect relatively subtle differences in visual perception, given the fact that the differences in stimulus durations was relatively small (17 ms vs. 50 ms).

## DISCUSSION

### STRENGTHS AND POTENTIAL APPLICATIONS OF SDR

The main novelty of SDR is that it is a method for numerically representing verbal descriptions of the ground truth (in the present case, the visual images). To our knowledge, methods to do this simply do not exist at present. Note that, reduced to its essentials, SDR requires only that the ground truth that the verbal account describes be available for independent evaluation. Given this simplicity of its requirements, SDR is potentially applicable to wide variety of potential real-world applications in which qualitative, verbal descriptions of real-world experiences need to be quantified (see below).

Our experimental results demonstrate that SDR is useful for quantifying qualitative reports of visual scenes. Although we illustrate the method by varying stimulus duration, we expect that the method should be applicable to any case in which subjective experience, visual or otherwise, are verbally reported, by normal subjects or patients, as long as the ground truth that elicited the experience can be independently evaluated. It also stands to reason that second-hand reports of percepts, such as a clinical provider’s verbal observations of the patient’s behavior, can be similarly, quantified using the same underlying principles.

Three main strengths of SDR are particularly worth noting. First, it places very few constraints on the patients (or subjects), in that it allows patients to view the stimuli freely and naturally, and describe their percepts in their own words. This allows the researcher, clinician or the machine learning algorithm to evaluate the subject/patient in a setting that is natural and minimally stressful. In this sense, our method is different from other methods of quantifying qualitative data, which generally require streamlining or formatting of the qualitative data, e.g., using questionnaires or forms (for reviews, see Udupa, 1999; Auerbach and Silverstein, 2003; Gustafson and McCandless, 2010; Sauro and Lewis, 2012; Bazeley, 2013). Second, SDR can, in principle, preserve much of the richness of the verbal reports, depending on the rules and algorithms used for evaluating the reports. Note also that the scores need not necessarily be integer rank scores; it should be possible, in principle, to develop algorithms for assigning fractional scores that treat the underlying descriptors as values of a continuous variable, rather than of a discrete or categorical variable. Third, as noted above, this method is likely to be flexible and versatile, with a broad array of potential applications, given that its requirements are ultimately minimal, *viz*., a verbal description and the ground truth that elicited the description. For this reason, SDR should be applicable to a wide variety of stimuli (including drawings, photographs, or videos, and non-visual stimuli such as sounds and haptic objects), the aspect of the stimulus perceived (such as some affective aspect of the stimulus, the texture of an object, the origin of a sound, etc.). Thus, a pollster using focus groups to evaluate the impact a political or commercial advertisement can use the same set of SDR principles as an ophthalmologist or a neurologist evaluating a patient’s deficits in one or more of the senses, an educator testing students or a recruiter testing the aptitude of the applicants to comprehend complex real-world situations.

It is worth noting that, as alluded to in the Results section, machine learning methods can be devised to carry out the aforementioned steps 2 (independent evaluation of the subjects’ reports) and 3 (*post hoc* statistical analyses of the evaluators’ reports) of SDR. This would make the given implementation of SDR more objective by removing the contribution of the evaluators’ subjectivity from the process. In addition, our method has potential applications to machine learning itself, because it allows machines to process language using a numerical representation thereof. To our knowledge, methods to do this do not currently exist either.

### SOME IMPORTANT CAVEATS AND POTENTIAL FUTURE IMPROVEMENTS

There are four caveats that are particularly important to note. First, as noted earlier, our results only provide a “proof of concept,” and do not, by themselves fully validate this method. In order to validate SDR, one needs to show that SDR independently produces essentially the same results as that obtained by a different, established method (for reviews, see Willig and Stainton-Rogers, 2008; Denzin and Lincoln, 2011; Lezak, 2012). SDR also needs to be standardized for each intended purpose. For instance, the conditions under which it yields the most reliable results for a given purpose (e.g., evaluating hemianopsia patients) remain to be delineated. The scoring rules also need to be further developed and standardized. Standardizing and cross-validating SDR will also help further delineate its strengths, weaknesses, potential applications, and limitations. Note that the fact that SDR needs to be developed and refined further before it can be used in real-world applications does not by itself undermine the value of the underlying concept. After all, test development is necessarily an iterative process; any testing method has to undergo the aforementioned development process ( Brennan et al., 2006; Downing and Haladyna, 2006; Phelps, 2007; Gregory, 2010).

Second, SDR is meant not to supplant, but rather to supplement, the existing qualitative and quantitative methods. This caveat is particularly important in view of the fact that this method is yet to be tested extensively, and its strengths and weaknesses empirically documented. Specifically, it should be noted that SDR is by no means a universally applicable method for quantifying for qualitative reports, especially in cases where the underlying descriptors may not be reliably rank-ordered, e.g., in educational research (Hartas, 2010; Torrance, 2010; Haghi and Rocci, 2013). Moreover, as alluded to in the Results section, a verbal report, however indirect, is a prerequisite of SDR.

Third, the numerical scores of the evaluators are meant to be used in statistical tests that compare the relative values, not the absolute values, of the scores, such as rank-order or rank-sum tests. This is because our tests do not correct for the criterion level of the individual evaluator, e.g., whether a given evaluator may tend to score the reports “generously.” Using the relative values of the scores tends to correct for this, although only to the extent that a given evaluator’s criterion remains unchanged across the relevant dataset. To correct for these criterion effects, and to obviate the need for rank-based statistics, one can average over a large number of randomly chosen evaluators. For instance, one can create a large database of reports and scores for each given set of stimuli that can be used as a reference distribution to correct for any deviations from the norm. Note, incidentally, that having such a database also obviates the need to carry out paired statistics or even two-sample statistics, because the researcher can always compare a given single sample, e.g., a given subject’s reports for a stimulus duration of 17 ms, against a standard reference distribution of reports for that duration.

Finally, SDR is based on the hierarchical nature of object percepts, and therefore is not currently suited to evaluate percepts that are not hierarchical. This is especially true of affective percepts. However, by using a reference distribution as outlined above, one can extend our method to the assessment of non-hierarchical percepts.

### RELATION TO PREVIOUS WORK

What is most novel about our approach is that it exploits the hierarchical organization of natural objects to generate an arbitrarily rich numeric representation of the reported visual percept. To the best of our knowledge, methods to do this simply do not exist at present. But other aspects of our method, including the use of independent evaluators, have been previously used in other studies of visual dysfunction as well as normal visual function (Miles and Huberman, 1994; Glozman, 1999; Poreh, 2000; Zipf-Williams et al., 2000; Joy et al., 2001; Ogden-Epker and Cullum, 2001; Fei-Fei et al., 2007). Having independent evaluators independently scoring the subjects reports is effective, because it tends to average out random variance among evaluators while leaving intact non-random variance – that is, using independent evaluators helps achieve a measure of objectivity by way of shared subjectivity (Hegdé, 2008).

In the ultimate analysis, the utility of SDR is that it provides a novel approach to grappling with the breathtaking complexity and richness of our subjective visual experience. In this regard, it is of great potential utility in research, clinical and machine vision contexts alike.

## Conflict of Interest Statement

The authors declare that the research was conducted in the absence of any commercial or financial relationships that could be construed as a potential conflict of interest.
